# Some Aspects of Nonbeverage Alcohol Consumption in the Former Soviet Union

**DOI:** 10.1155/2015/507391

**Published:** 2015-04-30

**Authors:** S. V. Jargin

**Affiliations:** Peoples' Friendship University of Russia, Clementovski Per 6-82, Moscow 115184, Russia

## Abstract

Toxicity of some legally sold alcoholic beverages has contributed to enhanced mortality in Russia since 1990. Widespread drunkenness during the early 1990s facilitated privatization of economy: workers and some intelligentsia did not oppose privatizations because of drunkenness and involvement in illegal activities. Apparently, alcohol consumption and heavy binge drinking have been decreasing in Russia since approximately the last decade. Exaggeration of alcohol-related problems tends to veil shortages of the health care system. There are motives to exaggerate consumption of nonbeverage alcohol in order to veil the problem of toxicity of some legally sold beverages. It is essential to distinguish between legally and illegally sold rather than between recorded and unrecorded alcohol because sales of poor-quality alcoholic beverages in legally operating shops and kiosks occurred generally with knowledge of authorities.

The study [1] was based on a survey performed in the Republic of Belarus, but the discussion pertains also to Russia, where the pattern of alcohol consumption has been largely similar. Evaluating the results of surveys and questionnaires, it should be taken into account that these research tools have been largely discredited in Russia since the 1990s by means of widespread solicitations to take part in different surveys, often asking for private information: in the streets, per telephone, and also by agents coming to private homes. The author is not well informed about conditions in Belarus, but it can be reasonably assumed that certain policies have been analogous in both countries; besides, intensive migration has probably contributed to a rapprochement. There is generally no sense of responsibility among people with regard to surveys; answers to the questionnaires can be biased, especially concerning such a delicate topic as alcohol consumption.

During the antialcohol campaign (1985–1988) in the former Soviet Union (SU), massive consumption of alcohol-containing technical fluids and perfumery was observed, for example, window cleaner, which caused up to severe intoxications. Considering the large scale of the window cleaner sales in some places, for example, in Siberia, it was knowingly tolerated by authorities. According to [2], about half of the cases of lethal intoxication by alcohol-containing fluids during the 1990s were caused in some areas by legally sold beverages, while in many lethal cases a relatively low blood alcohol concentration was found. In some papers discussing alcohol surrogates in the former SU [1], the readers' attention has been diverted to industrial spirits, alcohol-containing medicaments, perfumery, and samogon (moonshine, homemade alcohol). It should be commented that the relative vodka price (cheap vodka price/average salary) was approximately 5 times higher in 1985 prior to the antialcohol campaign in the former SU compared to the year 2010 in Russia [3]. In Belarus, the average salary in 1993 was approximately equal to 7.3 liters of vodka compared to 73.9 in 2010 [4]. High consumption of nonbeverage alcohol would be a priori improbable in conditions of the relatively low prices for legally sold alcoholic beverages and their easy availability. It is in accordance with the author's observations that drinking of alcohol-containing technical fluids and perfumery decreased abruptly after the failure of the antialcohol campaign in 1989; consumption of samogon has obviously decreased as well. Some inexpensive medicaments containing medicinal alcohol, such as alcoholic solution of boric acid, have been consumed until recently. There are obvious motives to exaggerate the problem of drinking of technical fluids and perfumery in order to veil toxicity of some legally sold beverages. Following the abolition of the state alcohol monopoly in 1992, the country was flooded by poor-quality alcohol [3, 4]. It is essential to distinguish between legally and illegally sold rather than recorded and unrecorded or “undocumented” [4] alcohol because sales of poor-quality beverages in legally operating shops and kiosks occurred generally with knowledge and sometimes under participation of authorities or their members. In this connection, it is incorrect to name falsified vodka “noncommercial” [1, 5] as it has been massively sold through legally operating shops. Exaggeration of consumption of nonbeverage alcohol shifts the responsibility for the poisonings onto the consumers who supposedly choose to drink it. Extensive but insufficiently substantiated discussions tend to befog the problem.

Alcohol abuse has been a major cause of premature mortality in Belarus and Russia, but its role has apparently been diminishing since the last decade or more; more details and references are in [6]. In the author's opinion, among the main causes of the relatively low life expectancy in Russia [7] are insufficient quality and availability of health care [8] and poor quality and toxicity of some alcoholic beverages [3]. This matter requires further research. In particular, results of toxicological analysis of some alcoholic beverages sold in shops during the last decades would be of interest.
